# Rapid and selective manipulation of milk fatty acid composition in mice through the maternal diet during lactation

**DOI:** 10.1017/jns.2015.13

**Published:** 2015-05-06

**Authors:** Annemarie Oosting, Henkjan J. Verkade, Diane Kegler, Bert J. M. van de Heijning, Eline M. van der Beek

**Affiliations:** 1 Nutricia Research, Utrecht, The Netherlands; 2 Department of Pediatric Gastroenterology and Hepatology, Beatrix Children's Hospital - University Medical Center Groningen, University of Groningen, Groningen, The Netherlands; 3 Nutricia Research, Singapore 138671, Singapore

**Keywords:** Dietary fat quality, Milk fatty acid composition, Mouse models, ALA, α-linolenic acid, ARA, arachidonic acid, CTRL, control, FA, fatty acid, LA, linoleic acid, LCP, long-chain PUFA, LowLA, low linoleic acid, MCFA, medium-chain fatty acid, *n*-3LCP, *n*-3 long-chain PUFA, PN , postnatal day

## Abstract

Dietary fatty acid (FA) composition in early postnatal life can modulate growth and development and later metabolic health. Investigating programming effects of early dietary FA manipulations in rodents may be stressful and complicated due to the need of artificial feeding techniques. It is largely unknown to what extent breast milk (BM) FA composition can be directly manipulated by the diet. We exposed dams to different dietary FA compositions from postnatal day (PN) 2 until PN28. Dams with litters were randomly assigned to control (CTRL), high-medium-chain FA (MCFA), low-linoleic acid (LowLA), high-*n*-3 long-chain PUFA (*n*-3LCP) or high-*n*-3LCP and MCFA (*n*-3LCP/MCFA) diets, and diets were continued after weaning until PN28. FA compositions were determined in feeds, milk and in erythrocytes. BM MCFA content was independent from dietary MCFA intake. In contrast, the LowLA diet reduced BM LA content by about 50 % compared with the CTRL diet at PN7. BM of dams fed the *n*-3LCP or *n*-3LCP/MCFA diet contained about 6-fold more *n*-3 LCP than BM of the dams fed the CTRL diet at PN7. These changes in milk FA composition established after 5 d of dietary exposure did not further change over the lactation period. At PN28, the erythrocyte FA composition of the male pups correlated with analysed milk FA profiles. In conclusion, manipulation of the diet of lactating mice can strongly and rapidly affect BM FA composition, in particular of *n*-6 LA and *n*-3 LCP. Our present findings will facilitate mechanistic studies on the programming of adult metabolic health by dietary FA in the early postnatal period via direct and selective manipulation of the maternal diet.

Infants derive dietary fatty acids (FA) from either human milk or infant milk formula. In this early period of life, dietary lipids are the main source of energy (about 50 % energy) and the main supplier of fat-soluble vitamins and the essential FA linoleic acid (LA; 18 : 2*n*-6) and α-linolenic acid (ALA; 18 : 3*n*-3). The latter FA are precursors for the long-chain PUFA (LCP; C20–C24) arachidonic acid (ARA; 20 : 4*n*-6), EPA (20 : 5*n*-3) and DHA (22 : 6*n*-3), constituents of biological membranes and precursors for eicosanoid biosynthesis^(^^1^^)^.

The FA composition of infant milk formula is constant and relatively uniform to adhere to (inter)national legislation^(^^2^^,^^3^^)^. In Europe, the LA content of the different infant formulas must range between 11 and 20 wt% of total FA according to the EU Commission directive. ALA should range between 1 and 4 wt% and the LA:ALA ratio between 5 and 15. The addition of LCP is recommended by most authorities including the European Union and WHO, but limits are currently set to maximal 1 wt% for *n*-3 LCP and 2 wt% *n*-6 LCP^(^^4^^)^. DHA content may not exceed ARA content^(^^4^^)^.

The FA composition of infant formulas has been based on analysis of the FA composition of human milk and, for LCP content, on data concerning infant LCP status and on functional outcome such as growth and visual acuity^(^^5^^)^. Interestingly, however, human milk FA composition and content are not very constant: human milk lipid content and FA composition are affected by maternal diet and body composition, stage of lactation (colostrum, transitional or mature milk), interval between feeds during 24 h and volume ingested per feed, and, finally, lipid content even changes during a single feed (fore- *v.* hind-milk)^(^^6^^–^^8^^)^. The variation of human milk lipid composition is related to the origin of milk FA. Milk FA can originate from recent dietary FA intake, mobilised from maternal adipose tissue depots, or synthesised endogenously in the liver (i.e. *de novo* lipogenesis, from glucose and other dietary precursors)^(^^9^^)^. FA up to a chain length of fourteen carbon atoms (C14) are largely synthesised *de novo* in the mammary gland^(^^10^^,^^11^^)^. Approximately 50 % of FA with a chain length of sixteen carbon atoms (C16) is synthesised in the mammary gland and 50 % is derived from dietary C16, mobilised from adipose tissue or synthesised in other tissues, specifically the liver. FA with a chain length of eighteen carbon atoms (C18) and longer are derived from circulating plasma lipids, mainly chylomicrons and VLDL, either originating from maternal fat stores or recent dietary intake^(^^9^^,^^12^^)^. Studies with stable isotopes indicate that up to approximately 30 % of the milk LA and LCP are of dietary origin^(^^12^^,^^13^^)^.

The marked increase in dietary LA and decrease in *n*-3 LCP intake over the last decades^(^^14^^,^^15^^)^ is reflected in human milk FA composition^(^^16^^)^ and translated to contemporary infant milk formula FA composition^(^^17^^)^. This increase in LA intake has been hypothesised to induce adverse nutritional programming during lactation, thereby contributing to the current high global obesity incidence^(^^16^^,^^18^^,^^19^^)^. In contrast, a high *n*-3 LCP exposure in early life has been considered beneficial for later-life body composition and metabolic phenotype development^(^^20^^–^^22^^)^. In other words, dietary FA composition in early postnatal life is considered to modulate growth and development and ultimately to affect later-life metabolic health^(^^18^^)^. Yet, proof of causal relationships of early FA intake on later metabolic life is still rather scarce.

In order to establish the role of different dietary FA in postnatal life on adult body composition and metabolic phenotype, it would be helpful to modulate dietary FA intake of pups, without inducing stress by artificial feeding. We reasoned that the most natural way to modulate dietary FA intake of pups would be via changing the milk FA composition. In the present study we aimed to establish whether and to what extent FA composition of the maternal diet during lactation affects FA composition of murine breast milk. In order to prevent the effects of the maternal diets on the pups mediated via the placenta, we exposed dams to diets with different FA compositions after delivery, i.e. from postnatal day (PN) 2 onwards. Using different diets fed to the dams, we could address the effects and kinetics of manipulation of medium-chain FA (MCFA), of LA and ALA, and of *n*-3 LCP on milk FA composition.

## Methods

### Animals and procedures

All experimental procedures were approved by the Animal Experimental Committee (DEC consult) and complied with the principles of good laboratory animal care, in line with the ARRIVE guidelines for animal experimentation. Mice were conventionally housed in a temperature- and humidity-controlled room (21 ± 2 °C and 50 ± 5 %, respectively) on a 12 h light–dark cycle with lights on at 06·00 hours. Food (American Institute of Nutrition (AIN)-93-compliant semi-synthetic chow) and water were available *ad libitum*.

Female multiparous C57/BL6 mice were obtained from the breeding facility of Harlan Laboratories (Horst) and mated with males of the same strain. Males were introduced in the home cage of two females and removed from the cage after 3 d. After 2 weeks, females were checked for pregnancy and housed individually. On PN2, the dams were assigned to one of the five experimental diets, each containing 10 wt% fat (Table 1; four or five litters per diet). Litters (five to ten pups per litter) were not culled and left undisturbed to assure sufficient milk yield for subsequent analyses. Milk samples were taken three times during the lactation period. Dams and pups were anaesthetised (isoflurane–N_2_O–O_2_) and killed with cervical dislocation after blood sampling through heart puncture at PN28. Blood samples were collected in K3-EDTA-coated 1 ml microtubes (Greiner Bio-one). Erythrocytes of male pups were obtained by centrifugation at 1350 ***g*** for 12 min at 4 °C (Biofuge fresco; Heraeus), supernatant fractions were removed and erythrocyte samples were stored at –80 °C until FA analysis. Samples were exclusively collected from male pups, because planned studies at our laboratory concerning metabolic programming by postnatal dietary lipids involved only long-term follow-up of the male offspring.

**Table 1.** Dietary fatty acid composition of the experimental diets (g/100 g fat)

		Diet				
FA composition	Abbreviation	CTRL	LowLA	MCFA	*n*-3 LCP	*n*-3 LCP/MCFA
Medium-chain fatty acids (8 : 0–12 : 0)	MCFA	13·6	15·7	21·5	8·6	21·5
Docosahexaenoic acid (22 : 6*n*-3)	DHA	0·0	0·0	0·0	5·0	5·0
Eicosapentaenoic acid (20 : 5*n*-3)	EPA	0·0	0·0	0·0	1·2	1·2
Arachidonic acid (20 : 4*n*-6)	ARA	0·0	0·0	0·0	0·3	0·3
Linoleic acid (18 : 2*n*-6)	LA	14·8	6·4	14·3	11·9	11·8
α-Linolenic acid (18 : 3*n*-3)	ALA	2·6	1·6	2·6	1·1	2·0
Saturated fatty acids (8 : 0–24 : 0)	SFA	38·8	41·8	38·8	36·3	36·4
Monounsaturated fatty acids (16 : 1–24 : 1)	MUFA	38·6	41·9	39·2	36·7	35·8
Polyunsaturated fatty acids (C18–C24)	PUFA	17·4	8·0	16·9	20·0	20·8
Long-chain PUFA (C20–C24)	LCP	0·0	0·0	0·0	6·5	6·5
	C18 *n*-6:*n*-3	5·7	4·1	5·5	11·1	5·9
	LCP *n*-6:*n*-3	0·0	0·0	0·0	0·04	0·04
	Total *n*-6	14·8	6·4	14·3	12·2	12·1
	Total *n*-3	2·6	1·6	2·6	7·8	8·7
	Total *n*-6:*n*-3	5·7	4·1	5·5	1·6	1·4

CTRL, control.

### Diets

Diets were AIN-93-compliant^(^^23^^)^ and hence comprised 18 wt% protein, 60 wt% carbohydrates, and 5 wt% cellulose. All diets contained 10 wt% fat. FA composition of the diets was based on the human diet distribution of SFA, MUFA and PUFA, with 42 wt% SFA, 41 wt% MUFA and 17 wt% PUFA (Table 1). FA composition of the experimental diets varied due to the use of different oil blends comprised of vegetable and fish oils. Litters were exposed to either a control (CTRL) diet, with a FA composition comparable with that of infant milk formula, a diet high in MCFA (8 : 0–12 : 0; 21 wt% of total FA, 55 % increase compared with CTRL), a diet with high-*n*-3 long-chain PUFA (*n*-3LCP; 5 wt% DHA of total FA, 1·2 wt% EPA of total FA; this diet also contained a low amount of the *n*-6 LCP ARA: 0·28 wt%), a diet with 57 % reduced LA (LowLA; 6·4 wt% of total FA) and a diet combining high *n*-3LCP and high MCFA (*n*-3LCP/MCFA). Table 1 shows the FA composition of the CTRL and experimental diets according to recipe as calculated by the Department of Processing and New Technologies of Nutricia R&D. Analysis of the feeds for FA composition did not show any significant deviations from calculated recipes.

### Milk collection

Milk samples (30–550 μl) were obtained three times during the second week of lactation from dams with litters consisting of five to ten pups: on PN7–9, PN10–12 and PN13–15. Dams were separated from their litters for at least 3 h; litters were kept warm on a temperature-controlled surface. At 10 min after a subcutaneous injection with 0·3 ml oxytocin (1 IU/ml; Eurovet Nederland), dams were milked using an adjusted human lactation pump. Milking occurred at a fixed time (between 10·00 and 12·00 hours) to avoid diurnal rhythm confounding, and took about 10 min, after which dams were returned to their litters. Milk samples were frozen (–80 °C) until analysis for FA composition.

### Fatty acid analysis

Lipid FA composition in milk and erythrocytes was determined after lipid extraction according to Bligh & Dyer^(^^24^^)^. Milk samples (10 μl) or erythrocytes (200 μl) were transferred to glass tubes, 1 ml EDTA (1 %) solution, 2·2 ml methanol and 1 ml dichloromethane were added and vortexed for at least 5 min. Subsequently, 1 ml EDTA solution and 1 ml dichloromethane were added and the tube was vortexed again for 5 min. Tubes were centrifuged at about 2000 ***g*** for 10 min. Subsequently, 400 μl of the bottom (dichloromethane) layer was collected and transferred to another, high-quality glass tube and evaporated. Upon addition of 2 ml methanol and 40 μl concentrated H_2_SO_4_, tubes were placed in a heating block at 100 °C for 1 h. To the cooled tubes 2 ml hexane and subsequently 0·5 ml 2·5 m-NaOH were added, whereupon tubes were vortexed for 2 min. The top layer (hexane) was transferred to a new tube and evaporated. Residues were reconstituted in 200 μl iso-octane and FA composition was analysed on a gas chromatograph equipped with a flame ionisation detector. Specific FA levels are expressed as percentage of total FA, calculated as AUC of known and identified GC peaks.

### Statistical analyses

All data are expressed as means with their standard errors. Statistical analyses were performed using SPSS 12·0·1 (SPSS Benelux). Repeated-measures ANOVA was performed to analyse effects of experimental diets on milk composition with time (PN7–9, PN10–12, PN13–15) as the within-subject factor and diet (CTRL, MCFA, *n*-3LCP, *n*-3LCP/MCFA and LowLA) as the between-subject factor. Effects of experimental diet on male pup erythrocyte FA were analysed by means of univariate ANOVA. *Post hoc* analyses of significant main diet effects and time × diet interactions were performed using multiple comparisons with Fisher's least significant difference correction.

## Results

### Change in milk fatty acid composition due to maternal diet

**Fig. 1.**
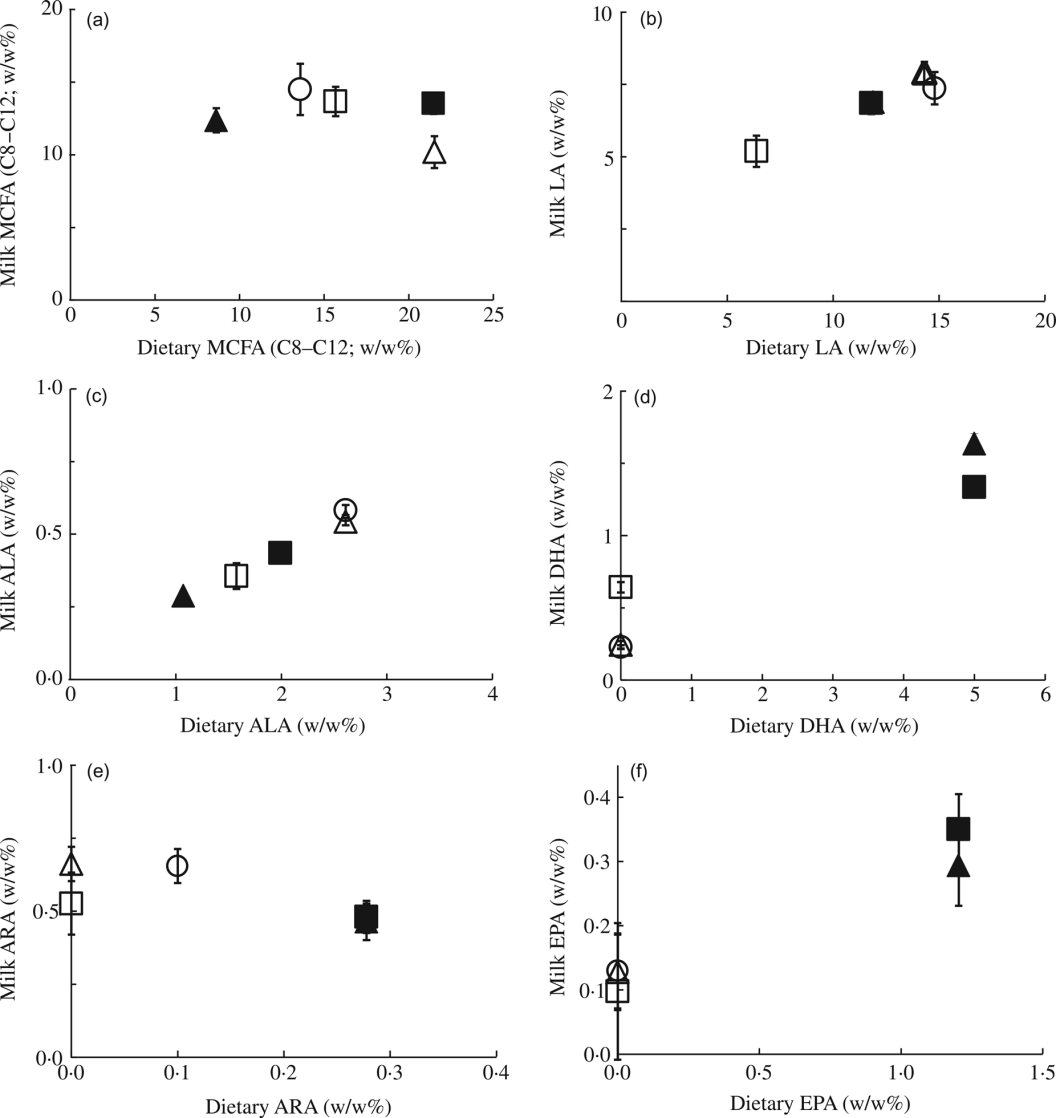
Effect of maternal dietary fatty acid (FA) intake in lactating mice on milk FA composition: correlations of mouse milk and dietary medium-chain FA (MCFA) (a); linoleic acid (LA) (b); α-linolenic acid (ALA) (c); DHA (d); EPA (e); and arachidonic acid (ARA) (f). Concentrations in milk at postnatal day (PN) 7–9 of dams fed a control (○), MCFA (Δ), *n*-3 long-chain PUFA (▲), *n*-3 long-chain PUFA/MCFA (█) or low-LA (☐) diet between PN2 and PN28. Concentrations are represented as wt% of total FA. Values are means (*n* 5 for all groups), with standard errors represented by vertical bars.

**Fig. 2.**
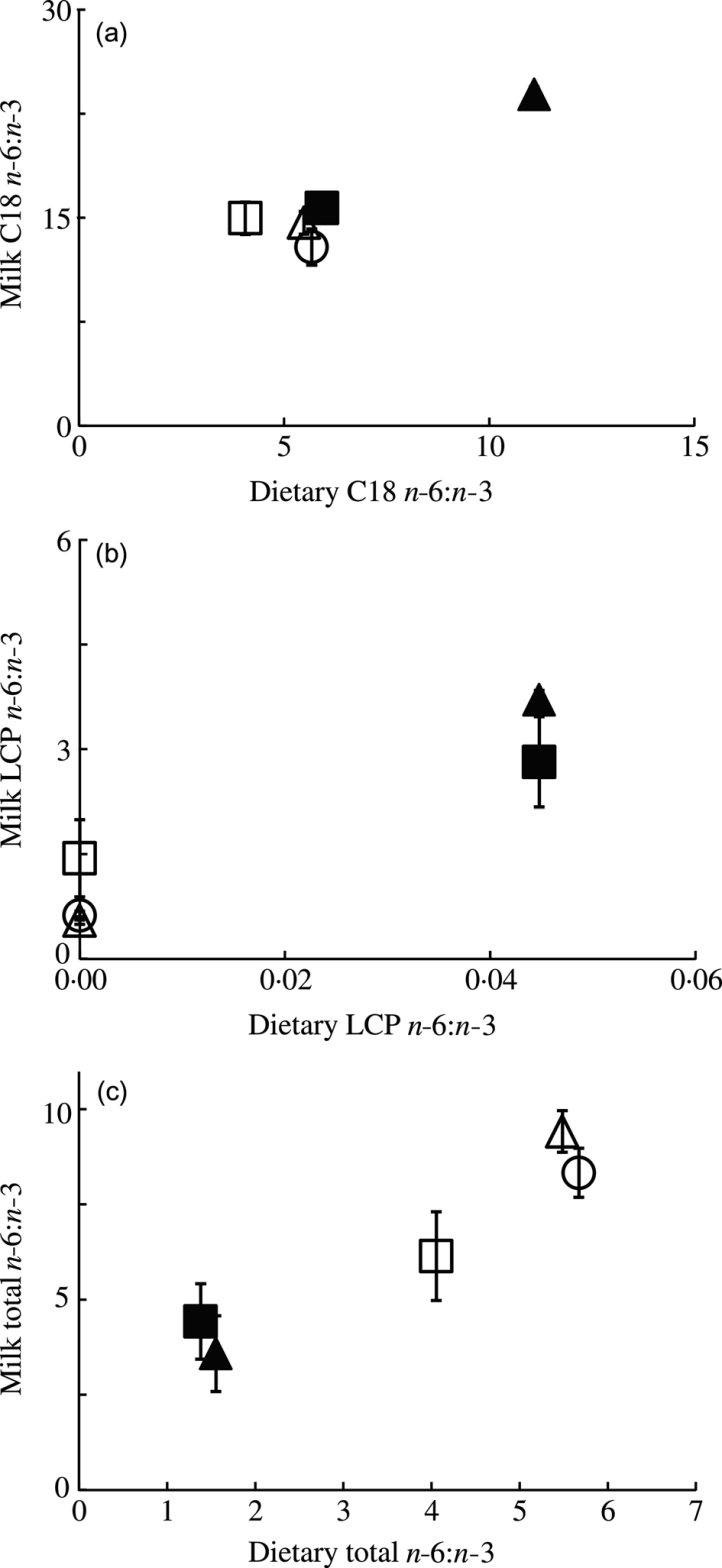
Effect of maternal dietary fatty acid (FA) intake in lactating mice on milk FA composition: correlations between linoleic acid:α-linolenic acid ratio (LA:ALA) (a); long-chain PUFA (LCP) *n*-6:*n*-3 ratio (b); and total *n*-6:*n*-3 ratio (c) in milk at postnatal day (PN) 7–9 compared with dietary ratios of dams fed a control (○), medium-chain FA (MCFA) (Δ), *n*-3 LCP (▲), *n*-3 LCP/MCFA (█) or low-LA (☐) diet between PN2 and PN28. Concentrations are represented as wt% of total FA. Values are means (*n* 5 for all groups), with standard errors represented by vertical bars.

Our primary aim was to determine to what extent FA composition of the maternal diet during lactation affects the milk FA composition in mice from PN7–9 onwards. Figs 1 and 2 depict the correlation between specific dietary and milk FA at PN7–9. Interestingly, increasing the dietary MCFA content by 50 % compared with the CTRL diet did not affect the milk MCFA content (Fig. 1(a)). The approximate 2·5-fold variation in dietary MCFA content for the dams (between about 8 and 22 wt%) was associated with a stable, around 15 % milk MCFA content. The results were quite different for LA and ALA. Milk LA closely reflected dietary LA content (Fig. 1(b)). The 57 % reduction of dietary LA in the LowLA group compared with the CTRL group (6·4 *v.* 14·8 wt%, respectively) resulted in a 45 % lower milk LA content (3·7 *v.* 6·8 wt% in the LowLA and CTRL groups, respectively; *P* < 0·001). Maternal ALA content also strongly influenced that of ALA in milk, in an apparent linear fashion (Fig. 1(c); *P* < 0·001). To address the effect of maternal LCP, we supplemented diets with 5 wt% DHA, 1·2 wt% EPA and 0·28 wt% ARA (the *n*-3LCP and *n*-3LCP/MCFA diet groups). The increase in DHA and EPA in the diet of the lactating dams corresponded with significantly higher levels of these FA in the milk. Milk DHA content increased by 35 % in dams fed *n*-3LCP and *n*-3LCP/MCFA, compared with dams fed either the CTRL, MCFA or LowLA diet (Fig. 1(d); *P* < 0·001). Interestingly, lowering LA increased milk DHA despite a concomitant decrease in ALA in this diet to obtain a LA:ALA ratio of 5 (*P* < 0·001). Milk EPA content even doubled, from 0·16 % to 0·33 %, in dams fed *n*-3LCP and *n*-3LCP/MCFA, compared with dams fed either the CTRL, MCFA or LowLA diet (*P* < 0·001, Fig. 1(f)). In contrast to the *n*-3 LCP DHA and EPA, a higher dietary content of the *n*-6 LCP ARA decreased milk ARA levels (Fig. 1(e)) from 0·67 % in the milk of CTRL dams compared with 0·52 % and 0·62 % in the milk of *n*-3LCP and *n*-3LCP/MCFA dams (*P* < 0·001). Milk ARA content was lower in LowLA dams (0·54 %) than in CTRL dams (0·67 %; *P* = 0·018).

The dietary C18 *n*-6:*n*-3 ratio was kept rather similar between experimental diets, except for an approximately twofold higher ratio in the *n*-3LCP-containing diet (Fig. 2(a)). The latter diet also strongly increased the C18 *n*-6:*n*-3 ratio in the milk FA (*P* < 0·001; Fig. 2(a)). The LCP *n*-6:*n*-3 ratio in milk was higher in the two LCP-containing diets, comparable with the results of the C18 *n*-6:*n*-3 ratio (Fig. 2(b)). The total *n*-6:*n*-3 ratio in the diets was manipulated more evenly across the experimental groups. Fig. 2(c) shows that the milk total *n*-6:*n*-3 ratios strongly (and linearly) correlated with those in the maternal diets.

### Time-dependent changes in milk fatty acid composition

The relative milk SFA content, which accounted for approximately 50 % of the total FA in all groups, increased by 5–10 % from about 48 % to about 55 % from PN7 to PN15 in all groups (*P* < 0·001; data not shown). The relative increase in SFA coincided with a slight, but significant, decrease in milk levels of MUFA (approximately 40 % of total FA) and PUFA (< 10 % of total FA; each –2 to –5 %, *P* < 0·001, data not shown). These effects were observed in all experimental groups, and thus seemed independent of the maternal dietary FA composition.

**Fig. 3.**
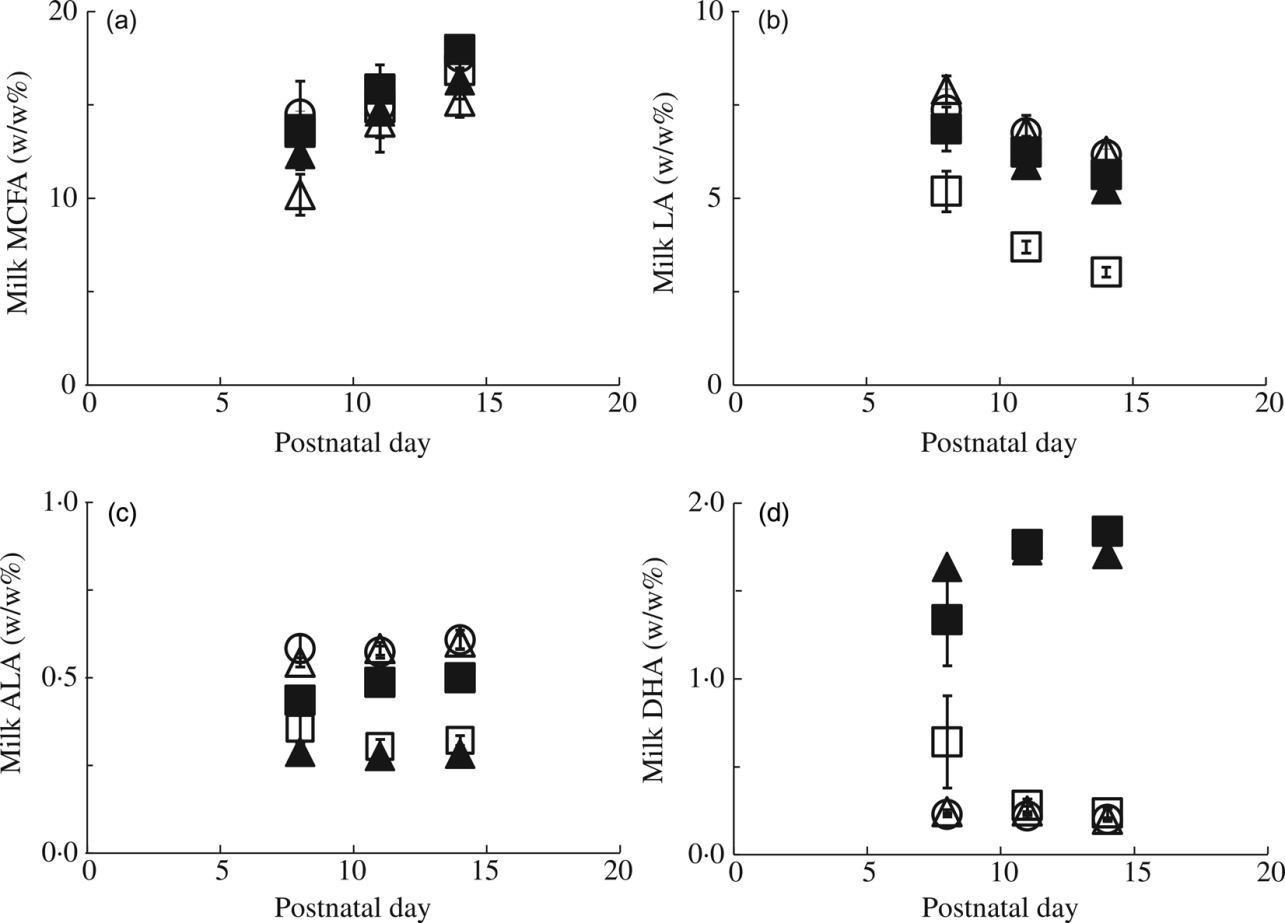
Changes in milk fatty acid (FA) composition over time in lactating mice fed different dietary FA composition. Milk medium-chain FA (MCFA) (a), linoleic acid (LA) (b), α-linolenic acid (ALA) (c) and DHA (d) concentrations during lactation (from postnatal day (PN) 7to PN15) of dams fed a control (○), MCFA (Δ), *n*-3 long-chain PUFA (LCP) (▲), *n*-3 LCP/MCFA (█) or low-LA (☐) diet between PN2 and PN28. Concentrations are represented as wt% of total FA. Values are means (*n* 5 for all groups), with standard errors represented by vertical bars.

In all experimental groups, milk MCFA increased significantly by 30 % between PN7–9 and PN13–15 (*P* < 0·001; Fig. 3(a)). In contrast, milk LA decreased in all groups between PN7 and PN15 (*P* < 0·001; Fig. 3(b)). Milk ALA content was rather constant over the time period studied (*P* = 0·333 l; Fig. 3(c)), whereas the kinetics of DHA were mixed (*P* = 0·881; Fig. 3(b)). Finally, milk EPA content remained constant from PN7 to PN15 (*P* = 0·633) and milk ARA decreased by about 28 % from PN7 to PN15 (*P* < 0·001; data not shown).

### Effect of milk and dietary fatty acid composition on erythrocyte fatty acid composition in pups

**Fig. 4.**
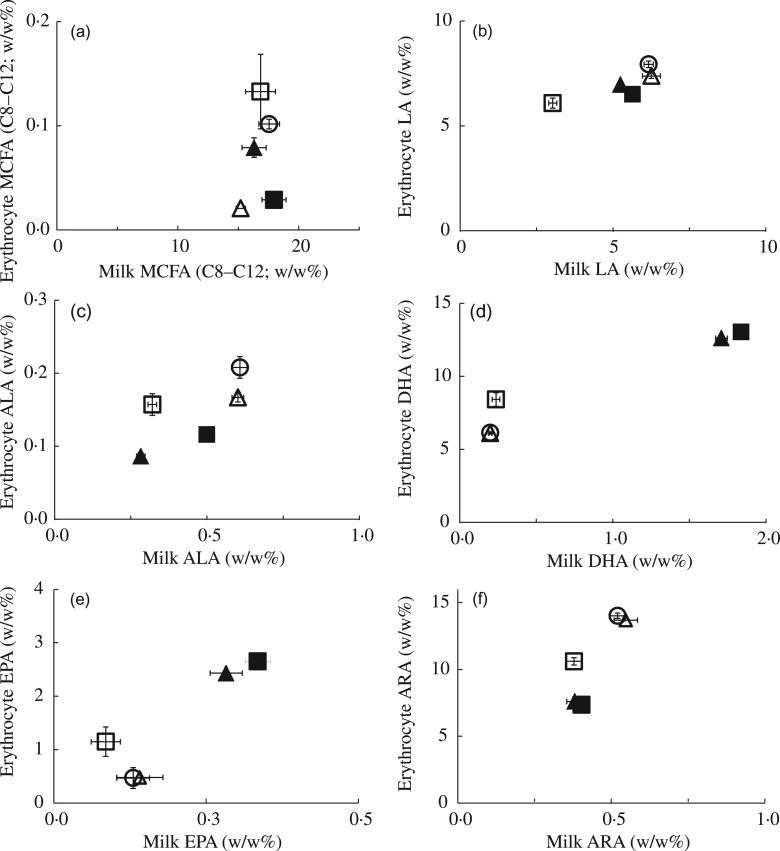
Effect of milk fatty acid (FA) composition during lactation on male pup FA status at weaning: medium-chain FA (MCFA) (a); linoleic acid (LA) (b); α-linolenic acid (ALA) (c); DHA (d); EPA (e); and arachidonic acid (ARA) (f) concentration of erythrocytes of male pups at postnatal day (PN) 28 (*n* 4–9) compared with milk MCFA, LA, ALA, DHA and ARA at PN13–15 (*n* 5) of dams fed a control (○), MCFA (Δ), *n*-3 long-chain PUFA (LCP) (▲), *n*-3 LCP/MCFA (█) or low-LA (☐) diet between PN2 and PN28. Concentrations are represented as wt% of total FA. Values are means, with standard errors represented by vertical bars.

**Fig. 5.**
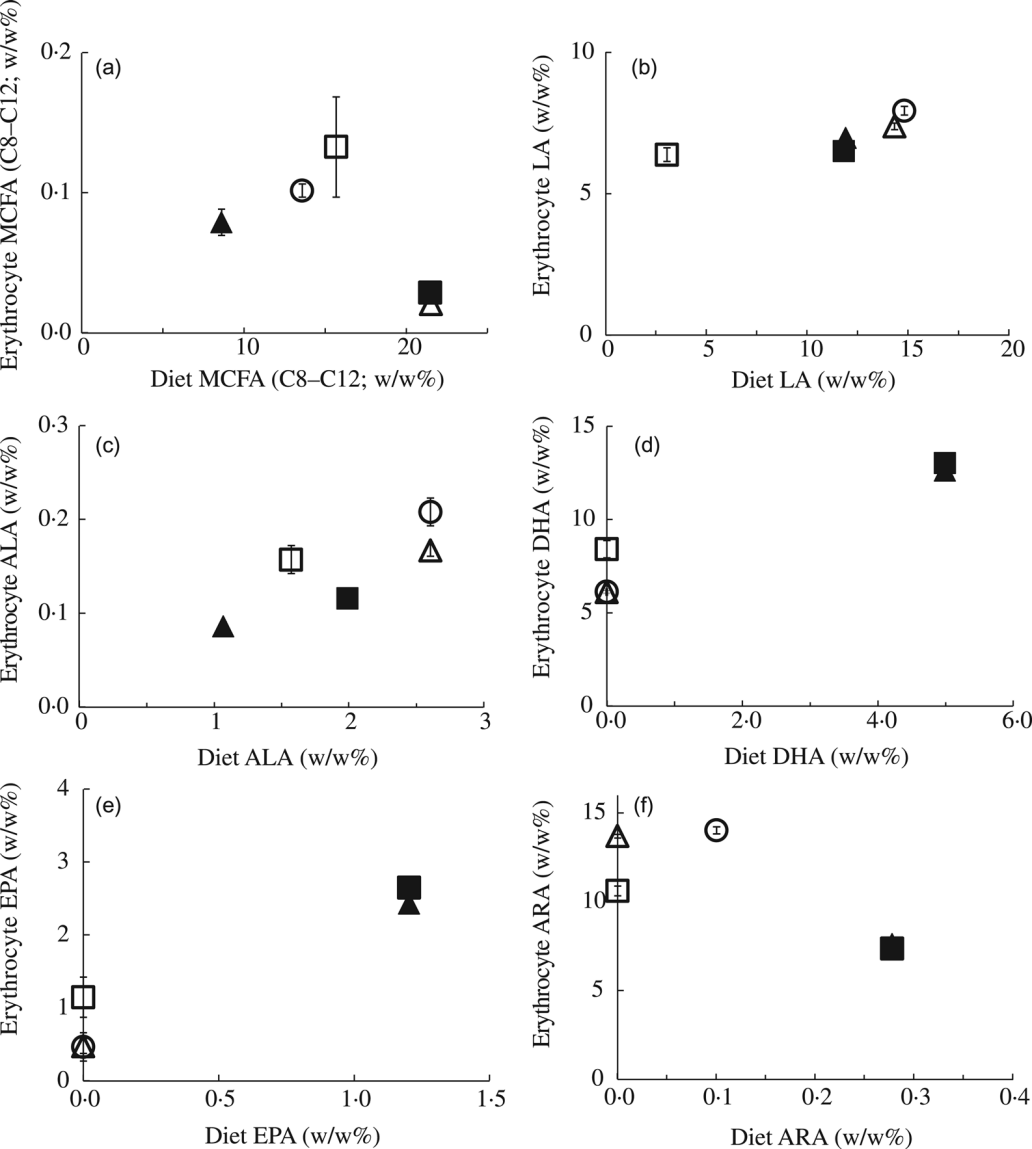
Effect of dietary fatty acid (FA) composition from postnatal day (PN) 2 to 28 on FA status of male pups at weaning: medium-chain FA (MCFA) (a); linoleic acid (LA) (b); α-linolenic acid (ALA) (c); DHA (d); EPA (e); and arachidonic acid (ARA) (f) concentrations of erythrocytes of male pups at PN28 (*n* 4–9) compared with dietary MCFA, LA, ALA, DHA, EPA and ARA of a control (○), MCFA (Δ), *n*-3 long-chain PUFA (LCP) (▲), *n*-3 LCP/MCFA (█) or low-LA (☐) diet fed to litters between PN2 and PN28. Concentrations are represented as wt% of total FA. Values are means, with standard errors represented by vertical bars.

Finally, we determined to what extent the milk composition at PN13–15 and the continued dietary manipulations after lactation from PN16 onwards influenced erythrocyte FA composition of the male pups at PN28 (Figs 4 and 5). The FA composition of the erythrocyte membrane consisted of about 45 % SFA, about 22 % MUFA and about 33 % PUFA for all experimental groups. Of the dietary FA that differed between the experimental diets, LA, DHA and ARA were the main erythrocyte membrane constituents with 6–8 %, 6–13 % and 7–14 % of total FA in the experimental groups, respectively. MCFA ( <0·1 %) and ALA (0·1–0·2 %) were barely incorporated in the erythrocyte membrane. Although to a lesser extent, the same applied for EPA: only 0·5–2·7 % of the total erythrocyte FA was composed of EPA. Comparable with the results on dietary and milk MCFA composition (Fig. 1(a)), erythrocyte MCFA content did not significantly correlate with milk MCFA (Fig. 4(a)), or with dietary MCFA (Fig. 5(a)). In contrast, the differences in milk LA (Fig. 4(b)) and in dietary LA (Fig. 5(b)) between the experimental groups were reflected in male pup erythrocyte LA content at PN28. Despite low absolute levels, similar correspondences were observed for ALA (Figs 4(c) and 5(c)). Erythrocyte DHA content correlated with the DHA content of milk and the maternal diet (Figs 4(d) and 5(d)) similarly as erythrocyte EPA content did (Figs 4(e) and 5(e)). Milk ARA content was partly reflected in erythrocyte ARA: CTRL and MCFA groups had a higher milk ARA compared with the LowLA group (Fig. 4(f)). However, the LowLA group had comparable milk ARA as the *n*-3LCP and *n*-3LCP/MCFA groups, whereas erythrocyte ARA was lower in the latter two groups (Fig. 4(f)). As stated above, dietary ARA was inversely correlated with erythrocyte ARA. Correspondingly, erythrocyte ARA was lower in *n*-3LCP and *n*-3LCP/MCFA pups concurrent with a higher dietary ARA content in these groups (Fig. 5(f)).

## Discussion

We aimed to determine whether changing the FA composition of the maternal diet during lactation in mice allows for a rapid and specific manipulation of milk FA composition and thus of the dietary FA supply to the pups. This approach would selectively change the FA quality in the early diet of mice in a non-stressful manner. Our data clearly show that in particular the LA, ALA and *n*-3 LCP content of the milk can be rapidly and specifically manipulated by the maternal diet composition (i.e. within 1 week), and further indicate that these short-term, relatively modest dietary changes are propagated into the erythrocyte FA composition of the pups after weaning. In contrast, milk MCFA content appears very resistant to manipulation of the dietary FA composition. These data clearly indicate that modulating dietary PUFA intake by newborn pups is feasible via postnatal alterations in the maternal diet and strongly support the concept that this approach can be used in mouse models to study nutritional programming.

The resistance of milk MCFA content against the manipulation of the dietary FA composition was evident across a wide range of maternal MCFA contents. The lack of effect of dietary MCFA manipulations are probably explained by the fact that milk MCFA are mainly synthesised *de novo* from carbohydrate and SCFA precursors^(^^10^^)^. Indeed, studies in rats^(^^25^^,^^26^^)^ and dairy cows^(^^27^^)^ have demonstrated that the dietary carbohydrate:lipid ratio determines MCFA content in milk: a higher carbohydrate content increases *de novo* MCFA synthesis in the mammary gland and reduces the uptake of longer-chain FA (LCFA; ≥ C18) from plasma, whereas a high-fat diet decreases mammary MCFA synthesis in rats and human subjects^(^^26^^–^^29^^)^. Novak & Innis^(^^30^^)^ have suggested that the availability of plasma LCFA determines MCFA synthesis, because lowering plasma TAG due to a low-fat or high-*n*-3LCP diet increases milk MCFA content in rats. Additionally, the FA composition of these high-fat diets influenced the extent by which MCFA synthesis was suppressed in rats; PUFA were more effective than MUFA whereas SFA were least effective^(^^31^^)^. In accordance, milk MCFA content in lactating women on a low-fat, high-carbohydrate diet was significantly higher than milk MCFA content of women on a high-fat, low-carbohydrate diet^(^^32^^)^. Indeed, MCFA biosynthesis pathways in the human mammary gland are similar to those in rodents^(^^10^^)^, but human MCFA synthesis is quantitatively low, probably related to the considerable higher lipid contribution in the human diet^(^^10^^,^^29^^)^. Our present observations indicate that investigating the role of early-life MCFA in the programming of later-life metabolic health in an animal model would either need artificial feeding of pups during lactation, an increase in the dietary carbohydrate:lipid ratio of the maternal diet during lactation, or would implicate a start of the dietary intervention after the lactation period. Modulating milk MCFA by exposure of lactating mice to a high-carbohydrate/low-fat *v.* low-carbohydrate/high-fat diet might be most effective. However, this change in dietary macronutrient composition might affect total lipid content of the milk^(^^33^^–^^35^^)^, although studies in rats showed that a low-fat/high-carbohydrate diet increased the percentage of MCFA, but did not affect total FA content of rat milk^(^^30^^,^^36^^)^.

In contrast to MCFA, modulation of dietary *n*-6 and *n*-3 FA in dams was highly effective in changing milk *n*-6 and *n*-3 FA content, including LA, ALA, DHA, EPA and (although negatively) ARA. These findings were in accordance with observational data in human volunteers, showing that 42 % of the variation observed in milk PUFA in the first month of lactation correlated with their variations in dietary PUFA intake^(^^37^^)^. In addition, a significant increase in milk ALA, LA, EPA and DHA was found in lactating women within 6 h after ingestion of a single bolus of various vegetable and fish oils, which correlated with the FA composition of the respective oils^(^^38^^)^. In our present study, lowering dietary LA effectively reduced milk LA and increased DHA. The latter might represent changes in the conversion rate of ALA to DHA due to lower dietary LA levels. Demmelmair *et al.*^(^^13^^,^^39^^)^ demonstrated that 23 to 30 % of milk LA was directly derived from dietary LA. A rat study with an experimental design comparable with our present study showed that dietary LA supplementation of rat dams from PN2 to PN15 increased milk LA at PN15^(^^40^^)^. Taken together these data suggest that the observation for translation of dietary LA levels in milk is rather generic and species independent.

The LowLA diet not only decreased LA content in the milk but also milk ARA content. This suggests that a quantitative part of the (maternal) dietary LA is metabolised before it is transferred as ARA into the milk. In contrast, studies with stable isotopes in human subjects indicate that the amount of milk ARA derived directly from LA synthesis is very limited^(^^6^^)^. Also, ARA content was comparable between women on a low-fat-diet *v.* an adequate-fat diet, and using a ^13^C-labelled LA tracer, only 0·01 % could be recovered from the milk ARA fraction, indicating that the majority of the milk ARA was obtained from pre-existing maternal fat stores^(^^41^^)^. Since rodents have a higher LCP biosynthesis capacity than human subjects^(^^31^^,^^42^^–^^44^^)^, this may explain the significant effect of low LA on milk ARA content in our mouse study.

Our data showed that supplementation of DHA and EPA resulted in a 6-fold increase in milk DHA and EPA compared with the CTRL diet. Milk DHA and EPA levels correspond with approximately 30 and 28 % of dietary DHA and EPA levels, respectively. These percentages are in accordance with human intervention studies showing that 30 % of milk LA and LCFA could be derived from dietary sources, whereas 60 % was derived from maternal lipid stores^(^^12^^,^^13^^)^. The 6 weeks of dietary supplementation of ALA and *n*-3 LCP during lactation, with similar LA and ARA content of the CTRL and supplemented diet, increased milk ALA and DHA and did not affect milk LA and ARA content^(^^45^^)^. Supplementation of 200 mg DHA to lactating women for 2 weeks doubled milk DHA content, without any effect on milk ARA. The use of a stable isotope tracer indicated that approximately 20 % of dietary DHA was secreted into the milk^(^^46^^)^.

In contrast to the C18 and the *n*-3 LCP FA, results were different for ARA. Feeding dams the *n*-3LCP or the *n*-3LCP/MCFA diet reduced milk ARA, compared with the maternal CTRL diet. This observation was counterintuitive, since the two experimental diets were supplemented with 0·28 % ARA, whereas the other diets did not contain any ARA. We speculate that this unexpected decrease in ARA may originate from the dietary DHA and EPA that were co-supplemented. Dietary DHA and EPA are known to decrease plasma and tissue ARA^(^^47^^)^, presumably because incorporation of *n*-6 and *n*-3 LCP in phospholipids depends on the dietary intake^(^^48^^)^. Also, dietary DHA and EPA inhibit Δ5 and Δ6 desaturase which inhibits the ARA synthesis from LA^(^^49^^)^. In order to determine whether the reduced ARA is indeed caused by the concurrent high dietary *n*-3 LCP, we would have to supplement ARA in an isolated fashion, which we did not investigate in the present study. Alternatively, the negative relationship between maternal diet ARA and milk ARA may be a species-specific effect. Increasing dietary ARA, despite high dietary DHA and EPA, resulted in increased milk ARA in lactating women, for instance^(^^50^^)^. These changes in diet and milk LCP content were also strongly correlated with maternal erythrocyte LCP content^(^^50^^)^.

The net balance of *n*-6 and *n*-3 PUFA in tissues of either dams and their offspring is determined by dietary intake of LA and ALA as well as of intake of their respective LCP, because LA and ALA depend on the same set of elongases and desaturases for conversion to their respective LCP, and because dietary LCP inhibit endogenous LCP synthesis. For instance, supplementation of ALA to a high-LA diet may have very limited effects on *n*-3 LCP status and metabolic health, because LA inhibits both *n*-3 LCP synthesis from ALA and incorporation in biological membranes^(^^16^^)^. To use the concept of maternal diet manipulation in mouse models for nutritional programming, it needs to be demonstrated that the dietary manipulation is propagated into the tissues of the growing pups. Indeed, dietary and milk FA compositions changed the erythrocyte FA composition of the male pups at PN28. Most evident effects of experimental maternal diet were found in DHA and ARA, and to a lesser extent in LA. We suggest that the explanation for this specificity relates to the fact that these PUFA are preferentially incorporated in membrane PL and are thus relatively abundant in biological membranes. The *n*-3 essential FA ALA is neither incorporated in membranes to a large extent, nor stored in adipose tissue depots. A considerable amount is oxidised to generate energy rather than being substrate for DHA and EPA conversion^(^^9^^)^.

In conclusion, our data show that short-term dietary manipulations of *n*-6 and *n*-3 essential FA and LCP are rapidly and specifically translated in maternal milk. These results indicate that modulation of PUFA supply to the pups during lactation by changing maternal dietary PUFA content is effective and can be used in mouse studies of nutritional programming. If the intention would be to investigate metabolic programming effects of MCFA, alternative dietary or artificial feeding methodologies seem warranted.

## References

[1] Innis SM (1992) Human milk and formula fatty acids. J Pediatr 120, S56–S61. 1343204 10.1016/s0022-3476(05)81237-5

[2] The Commission of the European Communities (2008) Consolidated version of Commission Directive 2006/141/EC of 28 October 2008 on infant formulae and follow-on formulae and amending Directive 1999/21/EC. 2006*L*0141*— EN—* 28.10.2008 *—* 001.001*—* 1. http://ec.europa.eu/food/food/labellingnutrition/children/formulae_en.htm

[3] Koletzko B, Baker S, Cleghorn G, (2005) Global standard for the composition of infant formula: recommendations of an ESPGHAN coordinated international expert group. J Pediatr Gastroenterol Nutr 41, 584–599. 16254515 10.1097/01.mpg.0000187817.38836.42

[4] Smit EN, Martini IA, Kemperman RF, (2003) Fatty acids in formulae for term infants: compliance of present recommendations with the actual human milk fatty acid composition of geographically different populations. Acta Paediatr 92, 790–796. 12892156

[5] Uauy R & Dangour AD (2009) Fat and fatty acid requirements and recommendations for infants of 0–2 years and children of 2–18 years. Ann Nutr Metab 55, 76–96. 19752537 10.1159/000228997

[6] Emmett PM & Rogers IS (1997) Properties of human milk and their relationship with maternal nutrition. Early Hum Dev 49, Suppl., S7–S28. 9363415 10.1016/s0378-3782(97)00051-0

[7] Mitoulas LR, Kent JC, Cox DB, (2002) Variation in fat, lactose and protein in human milk over 24 h and throughout the first year of lactation. Br J Nutr 88, 29–37. 12117425 10.1079/BJNBJN2002579

[8] Minda H, Kovacs A, Funke S, (2004) Changes of fatty acid composition of human milk during the first month of lactation: a day-to-day approach in the first week. Ann Nutr Metab 48, 202–209. 15256803 10.1159/000079821

[9] Innis SM (2007) Human milk: maternal dietary lipids and infant development. Proc Nutr Soc 66, 397–404. 17637092 10.1017/S0029665107005666

[10] Thompson BJ & Smith S (1985) Biosynthesis of fatty acids by lactating human breast epithelial cells: an evaluation of the contribution to the overall composition of human milk fat. Pediatr Res 19, 139–143. 3969307 10.1203/00006450-198501000-00036

[11] Hachey DL, Silber GH, Wong WW, (1989) Human lactation. II: Endogenous fatty acid synthesis by the mammary gland. Pediatr Res 25, 63–68. 2919120 10.1203/00006450-198901000-00015

[12] Hachey DL, Thomas MR, Emken EA, (1987) Human lactation: maternal transfer of dietary triglycerides labeled with stable isotopes. J Lipid Res 28, 1185–1192. 3681142

[13] Demmelmair H, Baumheuer M, Koletzko B, (1998) Metabolism of U^13^C-labeled linoleic acid in lactating women. J Lipid Res 39, 1389–1396. 9684741

[14] Sanders TA (2000) Polyunsaturated fatty acids in the food chain in Europe. Am J Clin Nutr 71, 176S–178S. 10617968 10.1093/ajcn/71.1.176s

[15] Wolmarans P (2009) Background paper on global trends in food production, intake and composition. Ann Nutr Metab 55, 244–272. 19752545 10.1159/000229005

[16] Gibson RA, Muhlhausler B & Makrides M (2011) Conversion of linoleic acid and α-linolenic acid to long-chain polyunsaturated fatty acids (LCPUFAs), with a focus on pregnancy, lactation and the first 2 years of life. Matern Child Nutr 7, Suppl. 2, 17–26. 21366864 10.1111/j.1740-8709.2011.00299.x PMC6860743

[17] Kuipers RS, Fokkema MR, Smit EN, (2005) High contents of both docosahexaenoic and arachidonic acids in milk of women consuming fish from lake Kitangiri (Tanzania): targets for infant formulae close to our ancient diet? Prostaglandins Leukot Essent Fatty Acids 72, 279–288. 15763440 10.1016/j.plefa.2004.12.001

[18] Innis SM (2011) Metabolic programming of long-term outcomes due to fatty acid nutrition in early life. Matern Child Nutr 7, Suppl. 2, 112–123. 21366871 10.1111/j.1740-8709.2011.00318.x PMC6860640

[19] Massiera F, Guesnet P & Ailhaud G (2006) The crucial role of dietary *n*-6 polyunsaturated fatty acids in excessive adipose tissue development: relationship to childhood obesity. Nestle Nutr Workshop Ser Pediatr Program 52, 235–242; discussion 243–245. 10.1159/000091076 16632969

[20] Hauner H, Brunner S & Amann-Gassner U (2013) The role of dietary fatty acids for early human adipose tissue growth. Am J Clin Nutr 98, 549S–555S. 23783299 10.3945/ajcn.112.040733

[21] Muhlhausler BS, Gibson RA & Makrides M (2010) Effect of long-chain polyunsaturated fatty acid supplementation during pregnancy or lactation on infant and child body composition: a systematic review. Am J Clin Nutr 92, 857–863. 20685946 10.3945/ajcn.2010.29495

[22] Muhlhausler BS, Gibson RA & Makrides M (2011) The effect of maternal omega-3 long-chain polyunsaturated fatty acid (*n*-3 LCPUFA) supplementation during pregnancy and/or lactation on body fat mass in the offspring: a systematic review of animal studies. Prostaglandins Leukot Essent Fatty Acids 85, 83–88. 21601438 10.1016/j.plefa.2011.04.027

[23] Reeves PG, Nielsen FH & Fahey GC (1993) AIN-93 purified diets for laboratory rodents: final report of the American Institute of Nutrition *ad hoc* writing committee on the reformulation of the AIN-76A rodent diet. J Nutr 123, 1939–1951. 8229312 10.1093/jn/123.11.1939

[24] Bligh EG & Dyer WJ (1959) A rapid method of total lipid extraction and purification. Can J Biochem Physiol 37, 911–917. 13671378 10.1139/o59-099

[25] Rodriguez-Cruz M, Sanchez R, Bernabe-Garcia M, (2009) Effect of dietary levels of corn oil on maternal arachidonic acid synthesis and fatty acid composition in lactating rats. Nutrition 25, 209–215. 18849148 10.1016/j.nut.2008.07.022

[26] Del Prado M, Villalpando S, Gordillo J, (1999) A high dietary lipid intake during pregnancy and lactation enhances mammary gland lipid uptake and lipoprotein lipase activity in rats. J Nutr 129, 1574–1578. 10419993 10.1093/jn/129.8.1574

[27] Glasser F, Ferlay A, Doreau M, (2008) Long-chain fatty acid metabolism in dairy cows: a meta-analysis of milk fatty acid yield in relation to duodenal flows and *de novo* synthesis. J Dairy Sci 91, 2771–2785. 18565935 10.3168/jds.2007-0383

[28] Neville MC & Picciano MF (1997) Regulation of milk lipid secretion and composition. Annu Rev Nutr 17, 159–183. 9240924 10.1146/annurev.nutr.17.1.159

[29] Barber MC, Clegg RA, Travers MT, (1997) Lipid metabolism in the lactating mammary gland. Biochim Biophys Acta 1347, 101–126. 9295156 10.1016/s0005-2760(97)00079-9

[30] Novak EM & Innis SM (2011) Impact of maternal dietary *n*-3 and *n*-6 fatty acids on milk medium-chain fatty acids and the implications for neonatal liver metabolism. Am J Physiol Endocrinol Metab 301, E807–E817. 21791621 10.1152/ajpendo.00225.2011

[31] Souza PF & Williamson DH (1993) Effects of feeding medium-chain triacylglycerols on maternal lipid metabolism and pup growth in lactating rats. Br J Nutr 69, 779–787. 8329353 10.1079/bjn19930078

[32] Nasser R, Stephen AM, Goh YK, (2010) The effect of a controlled manipulation of maternal dietary fat intake on medium and long chain fatty acids in human breast milk in Saskatoon, Canada. Int Breastfeed J 5, 3. 20170476 10.1186/1746-4358-5-3 PMC2838825

[33] Del Prado M, Delgado G & Villalpando S (1997) Maternal lipid intake during pregnancy and lactation alters milk composition and production and litter growth in rats. J Nutr 127, 458–462. 9082030 10.1093/jn/127.3.458

[34] Jensen RG (1996) The lipids in human milk. Prog Lipid Res 35, 53–92. 9039426 10.1016/0163-7827(95)00010-0

[35] Purcell RH, Sun B, Pass LL, (2011) Maternal stress and high-fat diet effect on maternal behavior, milk composition, and pup ingestive behavior. Physiol Behav 104, 474–479. 21605577 10.1016/j.physbeh.2011.05.012 PMC3142767

[36] Grigor MR, Geursen A, Sneyd MJ, (1984) Effect of chronic consumption of a high-fat diet on mammary metabolism. Int J Biochem 16, 691–694. 6381161 10.1016/0020-711x(84)90040-5

[37] Scopesi F, Ciangherotti S, Lantieri PB, (2001) Maternal dietary PUFAs intake and human milk content relationships during the first month of lactation. Clin Nutr 20, 393–397. 11534933 10.1054/clnu.2001.0464

[38] Francois CA, Connor SL, Wander RC, (1998) Acute effects of dietary fatty acids on the fatty acids of human milk. Am J Clin Nutr 67, 301–308. 9459379 10.1093/ajcn/67.2.301

[39] Demmelmair H, Baumheuer M, Koletzko B, (2001) Investigation of long-chain polyunsaturated fatty acid metabolism in lactating women by means of stable isotope techniques. Adv Exp Med Biol 501, 169–177. 11787680 10.1007/978-1-4615-1371-1_22

[40] Lien EL, Boyle FG, Yuhas RJ, (1994) Effect of maternal dietary arachidonic or linoleic acid on rat pup fatty acid profiles. Lipids 29, 53–59. 8139396 10.1007/BF02537091

[41] Del Prado M, Villalpando S, Elizondo A, (2001) Contribution of dietary and newly formed arachidonic acid to human milk lipids in women eating a low-fat diet. Am J Clin Nutr 74, 242–247. 11470727 10.1093/ajcn/74.2.242

[42] Blank C, Neumann MA, Makrides M, (2002) Optimizing DHA levels in piglets by lowering the linoleic acid to α-linolenic acid ratio. J Lipid Res 43, 1537–1543. 12235186 10.1194/jlr.m200152-jlr200

[43] Brenna JT, Salem N Jr, Sinclair AJ, (2009) α-Linolenic acid supplementation and conversion to *n*-3 long-chain polyunsaturated fatty acids in humans. Prostaglandins Leukot Essent Fatty Acids 80, 85–91. 19269799 10.1016/j.plefa.2009.01.004

[44] Burdge GC & Calder PC (2006) Dietary α-linolenic acid and health-related outcomes: a metabolic perspective. Nutr Res Rev 19, 26–52. 19079874 10.1079/NRR2005113

[45] Cherian G & Sim JS (1996) Changes in the breast milk fatty acids and plasma lipids of nursing mothers following consumption of *n*-3 polyunsaturated fatty acid enriched eggs. Nutrition 12, 8–12. 8838830 10.1016/0899-9007(95)00013-5

[46] Fidler N, Sauerwald T, Pohl A, (2000) Docosahexaenoic acid transfer into human milk after dietary supplementation: a randomized clinical trial. J Lipid Res 41, 1376–1383. 10974044

[47] Arterburn LM, Hall EB & Oken H (2006) Distribution, interconversion, and dose response of *n*-3 fatty acids in humans. Am J Clin Nutr 83, 1467S–1476S. 16841856 10.1093/ajcn/83.6.1467S

[48] Simopoulos AP (2008) The importance of the omega-6/omega-3 fatty acid ratio in cardiovascular disease and other chronic diseases. Exp Biol Med (Maywood) 233, 674–688. 18408140 10.3181/0711-MR-311

[49] Innis SM (1992) Plasma and red blood cell fatty acid values as indexes of essential fatty acids in the developing organs of infants fed with milk or formulas. J Pediatr 120, S78–S86. 1532829 10.1016/s0022-3476(05)81240-5

[50] Weseler AR, Dirix CE, Bruins MJ, (2008) Dietary arachidonic acid dose-dependently increases the arachidonic acid concentration in human milk. J Nutr 138, 2190–2197. 18936218 10.3945/jn.108.089318

